# Welcome to the 21st Century

**DOI:** 10.4103/2152-7806.63897

**Published:** 2010-05-31

**Authors:** James I Ausman

**Affiliations:** Editor-in-Chief, Surgical Neurology International

In the 21^st^ century, we live in a world of cell phones, instant worldwide information, television reporters embedded with advanced troops, Facebook, Twitter, failing world economies, global greed, illicit drugs, tattoos, multiple forms of music, divorce, remarriage, open homosexuality, terrorism, invasion of countries by immigrants, corrupt politicians and corporate executives, widespread lying, deceit and greed, false reporting in science, global warming and falsification of global warming data, widespread mortgage defaults, a desire for everything now regardless of cost, almost endless information, AIDS, poverty, a black President in the United States (USA), the USA's governmental and socialistic takeover of business, healthcare, and automobile industries (as in Europe, South America and other continents), the loss of manufacturing to cheaper labor in Asia, worldwide markets on eBay, a decline in moral values, lack of civic accountability and personal responsibility, and countless more changes that create the appearance of a world in chaos.

What is happening? Why are these things happening? We are all going through change, a change of such rapid speed and extent worldwide, that we are staggered by it.

We are at the beginning of a worldwide revolution for all people moving from the Industrial Age to the Knowledge Age, which is spreading over the world. Knowledge is more important than manufacturing. Manufacturing, as we all have seen, can be done cheaply in different parts of the world. However, those with knowledge have power, which those with manufacturing do not have. And that knowledge translates into wealth. And the more knowledge one has, the more creativity and ideas one can produce. This is what Alvin and Heidi Toffler, in their book *Revolutionary Wealth*, have written (Currency/Doubleday; 2006; New York.) They continue to describe the waves of change that have washed over mankind through history.

Over 200 generations ago, thousands of years ago, our ancestors were hunter-gatherers. They hunted and ate what they killed. They had no refrigerators; they did not live in large groups.

Then, 5,000-10,000 years ago, people began settling along rivers in China, the Middle East and Egypt, and began to farm and develop sub-specialized communities with specialized labor and jobs. These settlements were the beginning of civilizations, societies and the Agricultural Age. The family was dominant and the father was the head figure. The Agricultural Age existed until the 1600s with the beginning of the Renaissance and of the Age of Reason, which allowed concepts outside and independent of the teaching of the Church or authoritarian governments. From this newly-discovered knowledge, the development of machines led to the beginning of the Industrial Age. The Industrial Age opened the door to mass movement to cities, and the breakup of the traditional family and family values. In the 1900s, with the development of the automobile came the assembly line, and massification of everything for people in the cities, with mass media, mass marketing, products for the masses, and more.

But a new wave of change was flowing over civilization and that was the tremendous growth of knowledge that started in the 1600s. In the 1900s, creative ideas led to the computer, computed tomography and magnetic resonance scanning, decoding of the human gene, instant information via television, then through the Internet to the mobile phone. Individual expression replaced the loss of identity that occurred with massification in the Industrial Age. Now, we are entering into individual therapies for disease based on each person's genetic composition (see the section on Translational Neuroscience). Newspapers with preselected information by Editors are being replaced by individually selected information, which each person desires and can receive electronically. This is the Knowledge Age. No culture or civilization has ever experienced this rapid change as we are all now witnessing.

Yet, parts of Africa and the Middle East are still in the Hunter-Gatherer Age, while China and South American countries are moving from the Agricultural Age to the Industrial Age. North America is changing from the Industrial Age to the Knowledge Age economy. Europe is transitioning from the Agricultural, to the Industrial, to the Knowledge Age, depending upon the location. So, we live in a world with widespread differences in the lives of people. No wonder we have conflicts and wars and disruptions in relationships as one civilization cannot understand the changes of another. The 21st century will see more people moving up the ladder of change all over the world as the advances in knowledge affect everyone. This is life. It is not static. It is dynamically changing, constantly. If you have ever raised children, you learn that they grow faster than you can keep up with them. Their lives are not static.

In the past, people wrote on walls in caves or on parchment. Then people communicated verbally through the stories and songs of wandering minstrels. Not until the printing press was developed in China 2000 years ago and then again in Europe in the 1600s could large numbers of people share information. Newspapers and radio beamed this information to the masses in the 1900s, and then television in the 1950s. By the late 1900s, rocket-launched satellites allowed information to reach everyone in the world instantly. Remarkably however, scientific information is still mostly written in journals, which are available to only a limited number of scientists in the world. Those in the developing world cannot have access to this information.

Now with the Internet, there is the need to transmit the discoveries of science rapidly across the world so that everyone can have the latest information instantly. Is it a surprise that the major changes being experienced by societies worldwide would also affect the transmission of scientific information?

*Surgical Neurology International* is pioneering that change in neurosurgery and related neuroscience. It is leading the way into the 21^st^ century. Neurosurgeons and physicians worldwide have applauded the chance to read the latest science instantly, without having to pay large subscription fees, and to hear and see the worldwide expert speakers without leaving their home or country, also without cost. They want a chance to share experiences with their colleagues worldwide, which they will now have for the first time.

As a sign of the times, our journal is published in India. Publishing houses for traditional, print-based journals are on the decline, as more scientific journals become Open Access, or free, on the Internet. Transmission of instant information has replaced profit as the goal.

Yet, there is one caution to this explosion of worldwide knowledge and the huge amount of information available on the Internet: in his book, The Cult of *the Amateur*: *How Today's Internet is Killing our Culture*, (Doubleday, NY; 2007) Andrew Keen wrote that the problem with the information that is available to everyone is that there is no one to help select that which is valuable from that which is not. This is the fundamental problem with the Knowledge Age. How do you know what is true? What is valuable? *Surgical Neurology International* will solve that problem by having experienced neurosurgeons, neurologists, neuroscientists and others interpret this information for the reader to understand and to then decide personally what they think.

The fundamental problem with the 1960s' generation in the USA and worldwide is that it rejected the teachings of those over 30. You cannot discard the wisdom of 200 generations over centuries of human experience without consequences. It would be like rejecting all previous knowledge in science to take care of patients. Wisdom is having experience, knowledge, and good judgment. That is how the brain's neural networks are built and grow and how the right and left halves of the brain interrelate as we age. The young tend to use the left half of their brains. I have said many times that you can teach a monkey (technically) to operate or to use a computer but judgment-good judgment-is something that has to be taught and learned. That is what *Surgical Neurology International* is all about. It is not about masses of information, but about judgment. That will be the key to the Knowledge Revolution of the 21st century, and to wealth creation for all.

Welcome to the 21^st^ century. Welcome to *Surgical Neurology International*.

